# Combining Environment-Driven Adaptation and Task-Driven Optimisation in Evolutionary Robotics

**DOI:** 10.1371/journal.pone.0098466

**Published:** 2014-06-05

**Authors:** Evert Haasdijk, Nicolas Bredeche, A. E. Eiben

**Affiliations:** 1 Computer Science Department, VU University Amsterdam, Amsterdam, Netherlands; 2 Sorbonne Universités, UPMC Univ Paris 06, UMR 7222, ISIR, F-75005, Paris, France; 3 CNRS, UMR 7222, ISIR, F-75005, Paris, France; University of Sheffield, United Kingdom

## Abstract

Embodied evolutionary robotics is a sub-field of evolutionary robotics that employs evolutionary algorithms on the robotic hardware itself, during the operational period, i.e., in an on-line fashion. This enables robotic systems that continuously adapt, and are therefore capable of (re-)adjusting themselves to previously unknown or dynamically changing conditions autonomously, without human oversight. This paper addresses one of the major challenges that such systems face, viz. that the robots must satisfy two sets of requirements. Firstly, they must continue to operate reliably in their environment (viability), and secondly they must competently perform user-specified tasks (usefulness). The solution we propose exploits the fact that evolutionary methods have two basic selection mechanisms–survivor selection and parent selection. This allows evolution to tackle the two sets of requirements separately: survivor selection is driven by the environment and parent selection is based on task-performance. This idea is elaborated in the **M**ulti-**O**bjective a**N**d open-**E**nded **E**volution (monee) framework, which we experimentally validate. Experiments with robotic swarms of 100 simulated e-pucks show that monee does indeed promote task-driven behaviour without compromising environmental adaptation. We also investigate an extension of the parent selection process with a ‘market mechanism’ that can ensure equitable distribution of effort over multiple tasks, a particularly pressing issue if the environment promotes specialisation in single tasks.

## Introduction

The field of evolutionary robotics concerns itself with the use of evolutionary algorithms to design and optimise components of robotic systems, typically the robots’ controllers [1]. In most evolutionary robotics applications the evolutionary algorithm runs in a software simulator that captures the main features of the robot(s) and the environment and only the final solution as found by the evolutionary algorithm is transferred to real robotic hardware. Thus, the mainstream of evolutionary robotics uses evolution in an off-line fashion, before deployment, prior to the operational period of the robot(s); there is typically no further adaptation during the operational period.

Embodied evolutionary robotics is a sub-field of evolutionary robotics that implements evolutionary algorithms on the robotic hardware itself, during the operational period, i.e., in an on-line fashion. Running the evolutionary algorithm on the robots has substantial implications for the implementation of the evolutionary algorithm. Because evolutionary algorithms are population-based heuristics, embodied evolutionary robotics is often implemented in a group or swarm of robots: the evolving individuals (that represent robot controllers) are distributed over the swarm. Certain evolutionary operators imply exchange of information between robots (e.g., selection or crossover), while others could be executed internally by individual robots (e.g., fitness evaluation, mutation). Over the last 15 years, several embodied evolutionary robotics algorithms have been proposed to solve tasks of varying complexity, from goal finding and harvesting tasks to coordination behaviours [2]–[7]. An important benefit of embodied evolutionary robotics is that it offers the opportunity to acquire adequate behaviour on the fly, without the human in the loop. It enables robotic systems that are inherently adaptive, and therefore capable of (re-)adjusting themselves to previously unknown or dynamically changing conditions.

This paper addresses one of the major challenges such an adaptive robotic system has to overcome: the robots must satisfy two sets of requirements, one grounded in the environment they operate in (viability), the other defined by the tasks specified by the user (usefulness). In principle, evolution is a force capable of satisfying both of these requirements. Natural evolution has proven very successful at achieving viability: it generated life on earth through environmental selection [8]. Evolutionary algorithms, on the other hand, are very good optimisers driven by artificial fitness functions that define technical user objectives [9]. A similar dichotomy can be discerned between the areas of Artificial Life, where environmental adaptation is the dominating approach and traditional evolutionary robotics, where task-driven evolution is prevalent.

The field of evolutionary robotics is inherently task-minded, but to successfully learn how to tackle a task, the robots must survive (maintain battery level and structural integrity, avoid collisions, etc.) for long enough to do so. It is this challenge that this paper considers: how to combine environment-driven adaptation towards viability with task-driven optimisation towards usefulness.

Realising such a combination is not necessarily hard. Depending on the application scenario, there can be situations where viability and usefulness are aligned, that is, intrinsic environmental requirements are naturally met if the task is addressed. For example, the setup considered in Watson’s original work considers a target that everyone must find, thus ensuring successful genome exchange between robots, which is required for the algorithm to work, as a by-product of solving the task [2].

However, such an alignment between task-driven optimisation and environmental adaptation cannot be always assumed. In principle, these two priorities can be in conflict and pursuing increased taskperformance may lead to ‘self-destructive’ behaviour. In such situations special care is needed to formulate the task-related requirements. Using an implicit fitness function, rather than an explicit one, offers a way to relax (but not suppress) the selection pressure towards purely task-driven behaviour. An implicit fitness function evaluates the objective in terms of expected result rather than in the way that result was achieved [1]. Implicit fitness functions offer a less constrained setup to address a task: it can be very difficult a priori to define appropriate behaviour for a particular task while it is relatively easy to specify what the result of the evolving behaviour should be. Implicit fitness can be implemented in very different ways, such as favouring individuals which perform particular steering behaviours [4], or rewarding individuals with more energy when doing a particular task, such as during a harvesting task [10] or self-assembling [11].

Fitness functions, be they implicit or explicit, are the driving force in evolutionary robotics. This is a direct consequence of the fundamentally task-minded attitude where the main goal is to obtain useful robot behaviour and evolution is considered as an optimiser that helps to achieve this goal. This mindset is reflected in the generic algorithm setup that follows the logic of usual evolutionary algorithms [12]. In particular, parent selection and/or survivor selection (a.k.a. replacement) use fitness information. For the implementation of these selection operators there are various mechanisms in the literature. Some of these require a quantified assessment of fitness, e.g., roulette wheel selection, some only need a sorted population, e.g., rank-based selection, and some do not need quantitative fitness at all, only a comparison between candidate solutions, e.g., tournament selection. Nevertheless, they all work by assessing (relative) fitness before a choice is made and use this information to select an individual. Therefore, evolutionary robotics and usual evolutionary optimisation belong to the same type of evolutionary computing, where fitness is prime and the chances for reproduction and/or survival are derived from it.

This focus on optimising task performance can be counter-productive in certain situations, particularly in situations where the task requires behaviour that is at odds with the environment. In such situations, performing the task may carry a risk in terms of survival (e.g., robots fail to maintain battery levels because of the task focus) or in terms of procreation (e.g., robots stay out of communication range and so cannot exchange genetic material). When this is the case, purely task-focussed approaches from embodied evolutionary robotics may well fail because of their inability to abandon objectives while basic survival strategies are not found and maintained.

Of course, there is a considerable body of research where task performance plays no role whatsoever, where there is, indeed, no task at all. Here, reproductive ability and survival are the driving forces and fitness is a derivative of procreative success instead of the other way around. Evolution of the population solely depends on the ability of its individuals to spread copies of their genomes (i.e., to generate offspring), and some individuals turn out to be more successful than others at doing so. These individuals are increasingly apt at surviving and procreating in their environment. In particular, adaptation to the environment through open-ended evolution without any task at all has long been studied in artificial life [13] and yielded a variety of methods and algorithms such as TIERRA [14] and followers ([15]–[20], etc.). Such research investigates evolution as an adaptive process per se, driven by selection pressure that emerges from the interaction between individuals and their environment, just as it does in nature. In contrast to the fitness function-based comparison in evolutionary robotics, these methods do not actually compare individuals, but successful individuals can only be identified a posteriori, rather than selected a priori: the number of offspring determines whether a particular genome was successful rather than the other way round. Of course, this also means that beyond adaptation to the environment, evolution will not address any user-specified task.

The medea algorithm is an example of such a purely environment-driven evolutionary adaptation mechanisms in a collective robotics setup [21]. It considers a set of robots with limited communication capabilities. Evolution relies on the diffusion of genomes from peer to peer, and genomes compete with one another to spread over the population of robots. To do so, each robot carries a genome, which defines its behaviour during a predefined amount of time (its “lifetime”). Whenever two robots are close enough, each transmits a mutated copy of its current genome to the other, and store the incoming genome in a list for further use. At the end of the robot’s lifetime, the list of previously received genomes is emptied except for one arbitrarily selected genome, which then replaces the genome used so far. Because the new genome is arbitrarily selected, there is pressure towards genomes that are able to drive their “vehicles” (i.e., the robots) to spread themselves over the population, favouring behavioural strategies better fitted to the environment.

In this paper, we argue that balancing evolution between environment-driven adaptation and taskdriven optimisation represents a vital step towards implementing autonomous, functional, responsive and self-sufficient robot collectives that operate reliably in environments where human supervision is impossible, too expensive, or limited. To this end, we introduce monee (**M**ulti-**O**bjective a**N**d open-**E**nded **E**volution), a paradigm that allows the definition of an objective function without surrendering environment-driven adaptation. The main idea is to exploit the fact that evolutionary methods have two basic selection mechanisms and to use these in different roles: survivor selection driven by the environment (purely environment-driven, e.g., as described above for medea) and parent selection based on task-performance.

For the sake of generality, we assume that robot collectives will have multiple tasks, e.g. because the overall task consists of subtasks like monitoring, collecting and transporting resources. Jones and Mataríc [22] already noted that collectively tackling multiple tasks entails a division of effort: if there are multiple tasks, the population of robots as a whole must tackle all of them, i.e., the collective effort must be evenly distributed over all the tasks. The tasks and/or the environment may prevent generalisation at the individual level (e.g., it may not be possible to both collect and transport resources effectively), enforcing specialisation at the individual level, but the population as a whole must learn to perform all tasks simultaneously regardless.

To ensure that the collective effort is equitably distributed over all tasks when there are multiple tasks, we propose to introduce a market mechanism that regulates task-based rewards during mate selection according to the market logic that scarcity increases worth. The monee framework implements such a market mechanism: in our multiple task context this implies that tasks that only a few robots (can) perform yield relatively high rewards and therefore higher selection probabilities. Market-based schemes provide a well known solution to the task allocation problem in multi-agent and multi-robot settings, for instance in [23], [24]. Market-based parent selection in monee exploits this method to achieve multiobjective task-driven adaptation of robot behaviour.

In the following, we present our monee implementation in detail and experimentally evaluate it on a variation of an harvesting task. We show that monee does indeed promote task-driven behaviour without compromising environmental adaptation. In other words, given a scenario with some task(s) for the robots –and measurable task performance– the robots adapt their behaviour to perform the task(s) without losing the abilities required for environmentally defined success. We investigate the interplay of the two selection mechanisms and the resulting selection pressure.

We also show that the market mechanism is essential to ensure equitable distribution of effort over multiple tasks. That is, in a setting with multiple tasks, the population will learn and perform all tasks rather than focussing unduly on a subset. We investigate how this capability responds to environments that force robots to specialise in single tasks at the individual level and settings with disparate reward levels for the tasks.

## Methods

### Algorithm

We investigate a monee implementation that adds task-driven selection to the environment-driven evolution in the medea system. Bredeche et al. describe medea [25] as an open-ended evolutionary algorithm where autonomous robots move around an arena while continually broadcasting their genome over a short distance. Meanwhile, the robots also receive genomes from other robots that come within communication range. When a robot’s lifetime (which is fixed) expires, it randomly selects one of the received genomes, modifies that using mutation and starts a new life of broadcasting this new genome. This defines an environment where procreation relates to movement: genomes that cause the robot to move around a lot are spread at a much higher rate than genomes that cause their host to stand still. This selection pressure derives only from the way the environment defines the mechanics of transmitting genetic material: there is no objective function or explicit evaluation of individual controllers. In our experiments, we made a slight change to the basic medea set-up by adding an ‘egg’ phase. Now, the robots do not listen for genomes as they move about, but instead, when a genome expires, the host robots stops moving for a fixed amount of time while it listens for genomes from passing live robots that come within the limited communication range.

Monee extends this open-ended approach as follows. To add task-driven parent selection to this environment-driven evolutionary process, the robots can amass credits by performing tasks during their lifetime. For instance, a robot could get one credit for every piece of ore it collects, one for successfully transporting it to a cart, and so on. When multiple tasks are defined, the robots maintain separate counts for the credits awarded for each task, for instance one counter for the pieces of ore collected and another one for the number pieces transported. When a robot transmits its genome to an egg, it passes the current credit counts along with the genome and the egg uses that information to select a genome when it revives.

This scheme is reminiscent of parental investment, which has been investigated in artificial life settings, including experiments with robots [26]–[28]. In artificial life parental investment is often used to give the offspring a starting value of (virtual) energy [29]–[32] and a parent’s energy level is often linked to task performance (e.g., agents tasked with eating grass to gather energy [31]). The monee scheme, however, differs subtly but crucially from such parental investment schemes: a parent does not actually invest when impregnating an egg because the credits aren’t transferred but copied; there is no cost to the parent.

When the egg phase finishes, the robot compares the credits for each genome it has received. To enable this comparison across tasks, the egg calculates an exchange rate between tasks. This ensures that genomes that invest in tasks for which few credits are found overall (presumably hard tasks) are not eclipsed by genomes that favour easier tasks. The exchange rate is a linear weighting scheme that equalises credits based on the scarcity of credits for the different tasks. It is calculated as follows: let 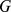 be the set of all genomes received by the egg and 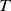 the set of all defined tasks. 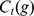 denotes the credits for task 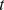 amassed with genome 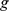. Then, the exchange rate for a task 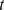 is: 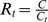, where 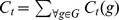 and 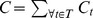. Genomes are then ranked according to the sum of credits multiplied by the exchange rates, 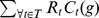, and a parent is selected using rank-based selection. Note that this scheme uses only local information: the calculations take only the credits earned by genomes received by this particular egg into account, as they were when the genome was transmitted. The exchange rate for the earnings per task implies that the more common credits are for a particular task, the less their worth and vice versa. Thus, parent selection becomes a marketplace for skills and features that the user requires.

The exchange rate’s reappraisal of task performance is similar to fitness sharing, a well-known technique that was introduced to promote genetic diversity and so prevent premature convergence in evolutionary algorithms. With fitness sharing, an individual’s fitness is reduced if there are many similar (in terms of their genetic makeup) individuals in the population. monee’s market mechanism is similar in the sense that it also reappraises fitness, disfavouring tasks that are more commonly tackled by robots in the population. A crucial difference with traditional fitness sharing is that monee considers an individual’s *behaviour*, not its genetic make-up (although syntactic fitness sharing in genetic programming shares this distinction [33]). Maybe more importantly, monee modifies fitness not to prevent premature convergence, but to ensure that the robot population tackles multiple tasks. Traditionally, fitness sharing is not necessarily associated with multiple objectives, but with maintaining diversity in general–typically, but not exclusively, in single-objective settings.

### Experiments

**Figure 1:**
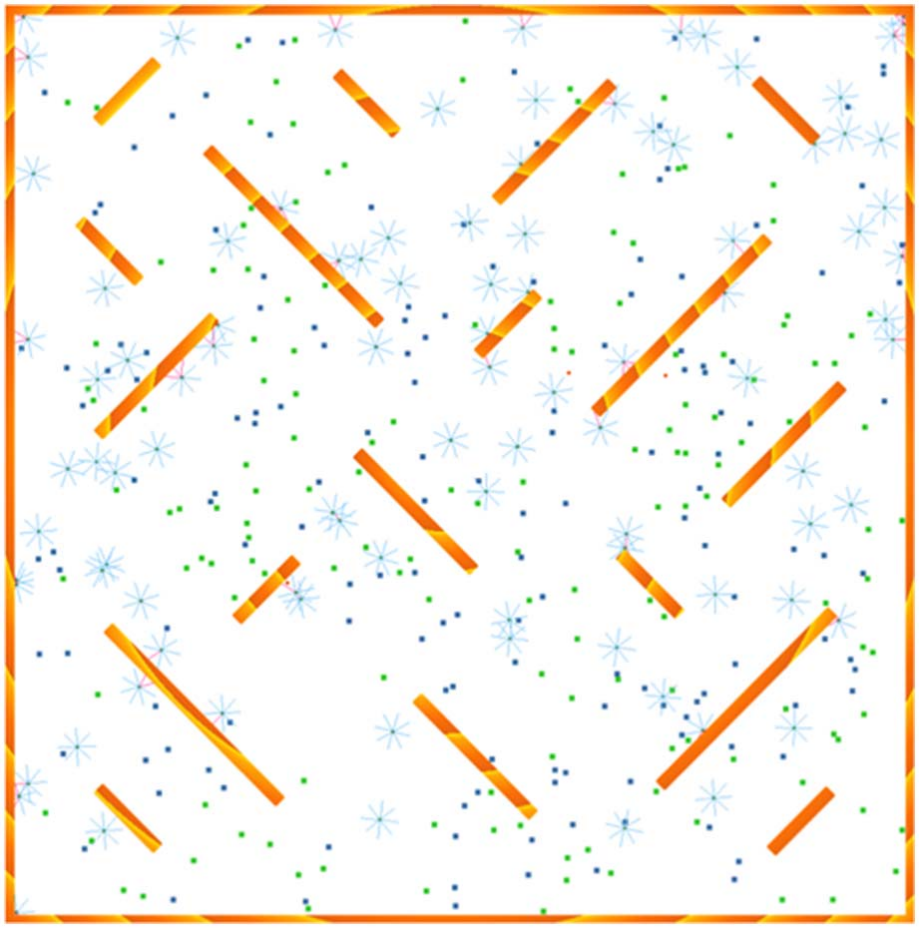
Experiment screenshot. Active robots are shown as small circles with sensor beams indicated, robots in egg state as red dots. Pucks are shown as small blue and green squares. The shaded orange rectangles indicate arena walls and obstacles.

We implemented the monee algorithm in a simple 2D simulator called RoboRobo [34], simulating 100 e-puck robots in an environment that contains obstacles and pucks. The sides of the square arena are roughly 330 robot body lengths long (1024 pixels in the simulator), and it contains a number of obstacles (see Fig. 1). We run 64 repetitions of the experiment and of all variations that we describe later.

There are two types of puck: green and red, defining a concurrent foraging scenario. Concurrent foraging is a variation of regular foraging where the arena is populated by multiple types of objects to be collected [22], rather than just a single resource. In our case, these objects are green and red pucks and the collection of each different colour is a different task. The pucks are spread throughout the arena, and they are immediately replaced in a random location when picked up. The robots move around the arena, spreading their genome as they encounter eggs and dying when their allotted time has passed. They collect pucks simply by driving over them; the more pucks they gather, the more likely their genome is to be selected once an egg they impregnated revives.

To detect pucks, the robots have 16 sensors that detect either red or green pucks (i.e., 8 sensors per puck-type). Each set of 8 sensors is laid out in the same manner as the standard e-puck infrared sensors: 6 face forward, 2 face to the rear. Because individual puck sensors only detect a single type of puck, collecting one type of puck is a task distinct from (but very similar to) collecting another type of puck. Thus, behaviour to collect either type of puck has to evolve separately. Each robot is controlled by a single-layer feed forward neural network which controls its left and right wheels. The inputs for the neural network are the robot’s puck and obstacle sensors. The robot’s genome directly encodes the neural network’s weights (3 types of sensor
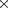
8 sensors
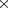
2 outputs plus 2 bias connections plus 4 feedback (current speed and current rotation to either output)  = 54 weights) as an array of reals.

As mentioned, the robots alternate between periods of active puck gathering (life phase) and motionless genome reception (egg phase). To prevent synchronised cycles among the robots, we add a small random number to each robot’s fixed lifetime. This desynchronises switching between life and rebirth even though our runs start with all robots perfectly in sync at the first time-step of their lifetime.

**Figure 2:**
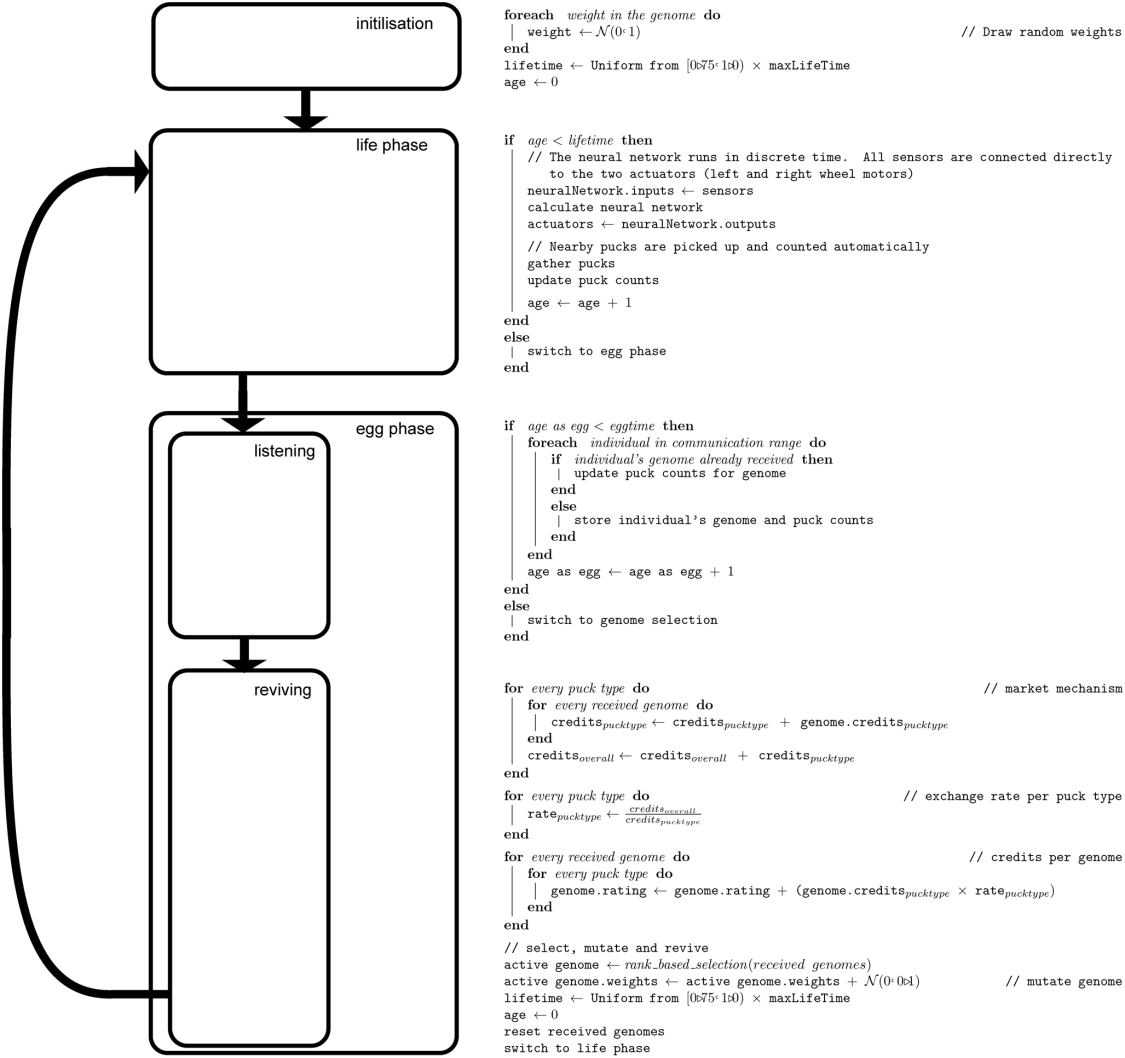
Robot flow of control in the experiments. To the left a flowchart indicates the life phases, to the right pseudo-code describing each phase in detail.

At the end of the egg phase, offspring is created by selecting a parent from the received genomes on the basis of their earnings and mutating the weights in that genome using gaussian perturbation with a single, fixed mutation step size 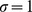. This single-parent, mutation-only scheme is common in evolution strategies that are known to perform well on problems with continuous-valued genomes [35]. Fig. 2.

Note that monee does not prescribe any particular controller implementation nor any choice of variation operator. The implementation we chose here of an artificial neural network with the weights encoded as real-valued genes provide a convenient, flexible and well-established representation. Quantitative analysis of initial trials with other controller formalisms showed very similar results. Therefore we will focus on the dynamics of the evolutionary dynamics rather than providing in-depth analysis of the particular internal properties of the evolved neural networks.

Fig. 2 depicts the flow of robot control in our experiments, Table 1 details the experimental set-up.

**Table 1:** Experimental set-up.

Experiment	
Number of robots	100
Simulation length	1,000,000 time-steps
Number of repeats	64
Number of pucks	150 green, 150 red, immediately replaced in a random location when picked up
Arena	See Fig. 1
**Controller**	
Controller	Feed forward neural net with 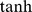 activation function
Input nodes	8 obstacle sensors, 16 puck detectors, 2 bias and 2 recurrent nodes
Output nodes	2 (left and right motor values)
**Evolution**	
Representation	Real valued vectors
Chromosome length	54
Initial weight distribution	Randomly drawn from 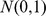 distribution
Mutation	Gaussian 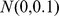 perturbation
Parent selection	Rank-based
Robot lifetime	2000 time-steps
Egg-phase duration	200 time-steps
Communication range	ca. 9 body lengths
Crossover	none

The standard settings for experiments reported in this paper. Some experiments vary one or more of these values as indicated in the experiment description.

### Simulation Software

The Roborobo code was written in C++. The code used for the experiments described here, together with settings files and scripts for the analysis of the results is available at http://www.few.vu.nl/~ehaasdi/papers/MONEE-PLOS/. We ran our experiments on the Lisa cluster at the Surf Sara facility, which consists mainly of 2.26 GHz nodes with Intel L5520 processors. Running on a single core, a typical single run would complete in just under 8 minutes.

### Statistical Tests

To quantify selection pressure from environment as well as task in various settings, we propose a measure that calculates the likelihood of random associations between behaviour and number of offspring in a population. We consider the distance covered, pucks collected and offspring count over the lifetime of the robots in the population. We split these individuals into classes with and without offspring and we split them along the median distance travelled or the median number of pucks collected during their lifetime. This results in two 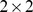 contingency tables: one relating offspring and distance travelled and one relating offspring to number of pucks collected. The cells of the contingency tables contain the count of individuals for that cell (e.g., the number of individuals with offspring and below median distance travelled). Fisher’s exact test is an appropriate test to determine the certainty of nonramdom associations between the categories in such contingency tables [36]. The test estimates the likelihood that the two classes in each contingency table (having offspring and above/below median distance travelled or pucks collected, respectively) are associated. The p-values resulting from these tests indicate the probability that there is no relationship between having offspring and having above- or below-median distance travelled or pucks collected. Thus, low p-values indicate high selection pressure and vice versa.

As mentioned above, we calculate the level of specialisation at population level to determine to what extent both tasks are taken up in equal measure by the robot collective. For each experiment, we calculate this ratio over all the pucks collected in the last 1,000 time steps of that simulation: it is the ratio of green pucks collected to all pucks collected: a ratio of 0.5 indicates that both types of puck were collected equally. When robots collect almost exclusively green pucks, this ratio will be close to 1.0, if the robots do not collect any green pucks, this ratio will be close to 0.0. To compare the ratio for different settings (e.g, with and without the market mechanism enabled), we compare the distributions of puck ratios for all runs with each setting. To test for statistical significance, we use a two-sample Kolmogorov-Smirnov test that compares the distributions of green-puck ratios for two sets of runs.

## Results and Discussion

### Adaptation to Environment and Task

To assess the take-up of the defined tasks, we need to establish whether the robots actually learn to gather pucks and whether they learn to cope with the environmental requirements under the monee regime. We compare the level of environmental and task-related adaptation through a control experiment where, as in medea, only the environment determines a genome’s chances of being selected: the number of pucks collected has no influence whatsoever. In other words, when an egg revives in the control experiment, it randomly selects one of the received genomes as parent for its new controller. As an additional control, we ran experiments with the market mechanism disabled: parents were selected on the basis of their total number of credits (pucks collected, in our case) without regard for their relative rarity.

**Figure 3:**
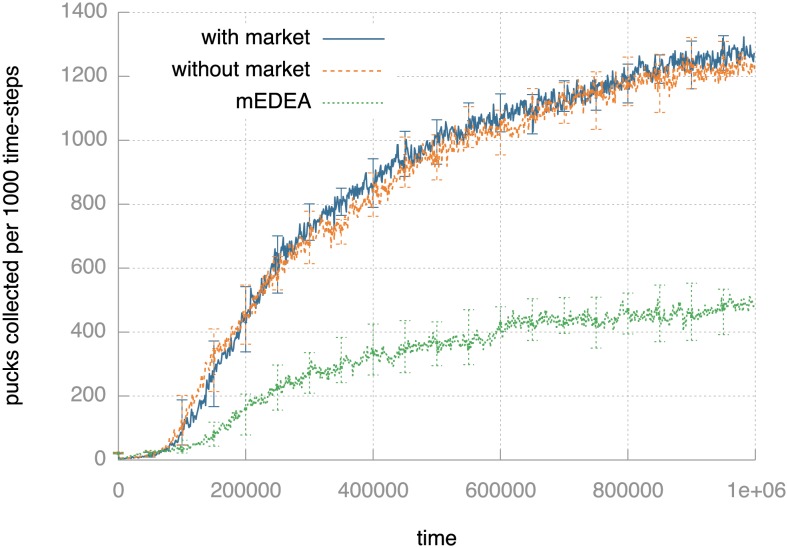
Task performance measured as the median number of pucks collected by the population per 1,000 time-steps. Plots show results for monee with and without market mechanism and with random parent selection (i.e., without any referral to the number of pucks collected–medea). The vertical bars indicate the 95% confidence interval for the medians. The robots clearly adapt behaviour to collect pucks. The number of pucks collected barely differs whether the market mechanism is in force or not. With random parent selection, the robots gather far fewer pucks: collection is a result of accidentally running over them during random movement.

Figure 3 shows clearly that populations with monee do learn to tackle pucks increasingly well after a brief initial phase. Until ca. 100,000 ticks, few pucks are collected, but then the number of pucks collected increases rapidly. The rate of increase starts to drop off towards the end of the runs, but the number is still increasing. There is no appreciable difference in the number of pucks collected with and without the market mechanism. As expected, the baseline algorithm (medea, where the number of pucks collected has no influence on parent selection) collects far fewer pucks – there is no pressure to adapt behaviour to collect pucks and they are only collected accidentally while moving about to spread genomes. The number of pucks collected with monee is much higher than would be expected solely due to random chance (i.e., with medea), and is therefore a driven rather than a passive trend.

**Figure 4:**
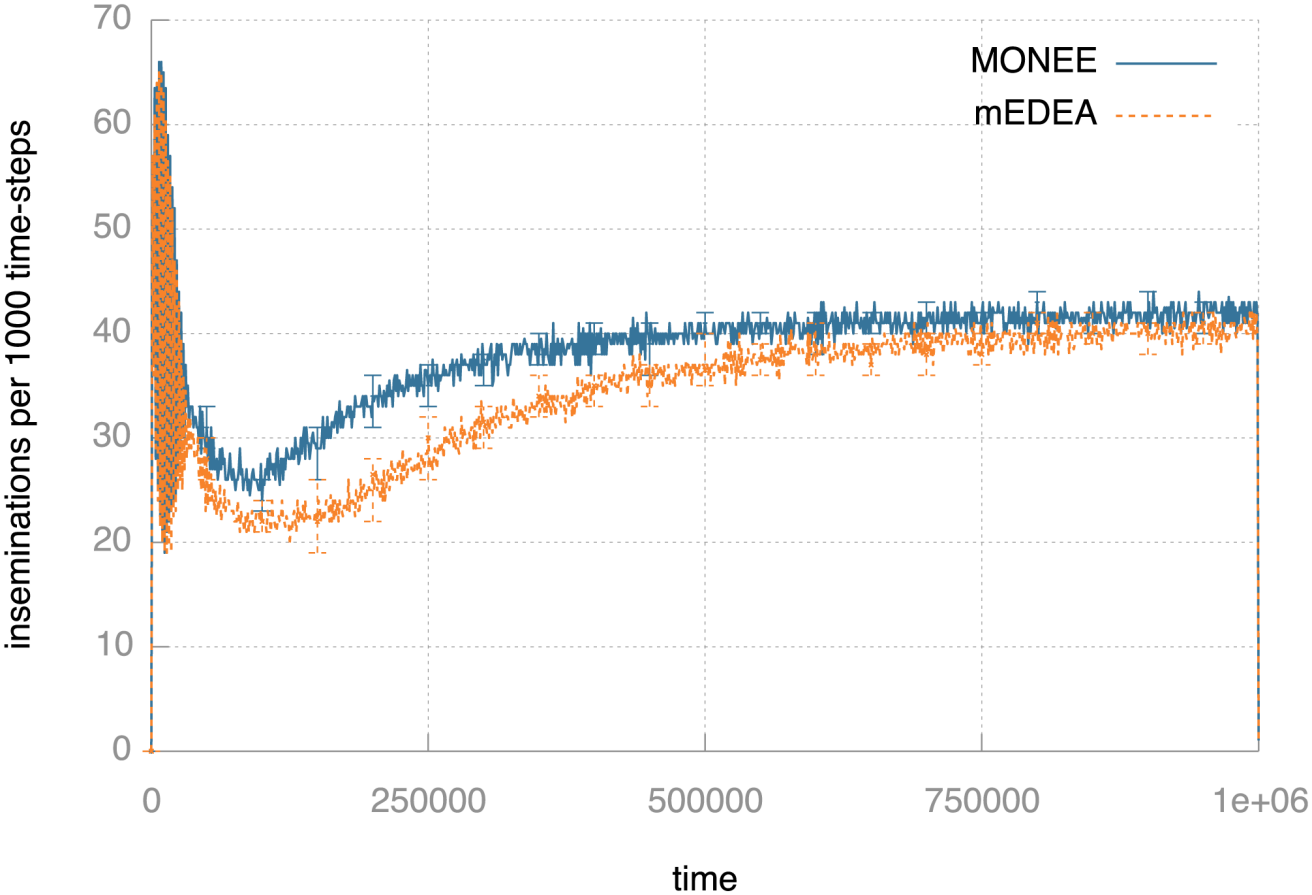
Viability measured as the median number of egg inseminations per 1,000 time-steps with monee and with random parent selection (medea). The vertical bars indicate the 95% confidence interval for the medians. Both curves indicate successful adaptation to the environment as robots become increasingly adept at spreading the genomes.

As a measure of adaptation to the environment, we count the number of genomes received by eggs (*inseminations*), excluding duplicate transmissions. Figure 4 shows the median number of inseminations per 1,000 time-steps for monee and for the baseline medea implementation. The initial peak is caused by the fact that the robots are concentrated in a small part of the arena at the outset of the experiments. As the robots spread out over the available area, the number of inseminations first decreases and then recovers as the robots adapt their behaviour. This increase is slightly faster with monee than with medea, but both level off at ca. 40 inseminations per 1,000 ticks. Overall, there seems to be little difference between vanilla medea and monee in terms of this measure of environmental adaptation.

### Investigating Selection Pressure

**Figure 5:**
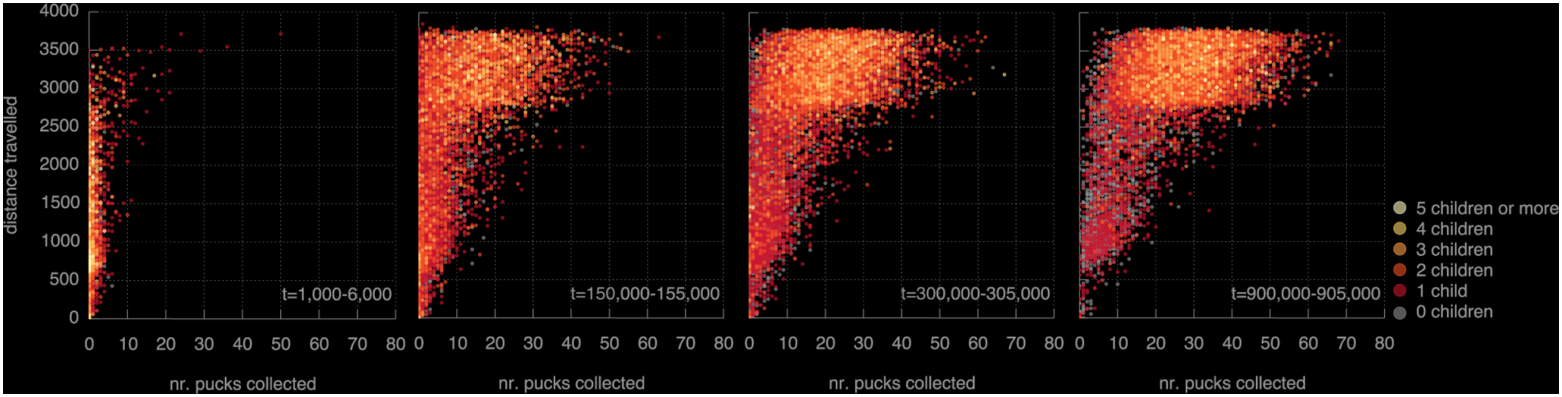
Offspring count vs distance travelled and number of pucks collected. From left to right, plots shown for time intervals at 1,000, 150,000, 300,000 and 900,000 ticks. Initially, distance travelled and number of pucks collected have little influence on an individual’s fecundity. As time progresses, the influence of travelling distances and collecting pucks becomes progressively pronounced.

There are two obvious determinants of selection pressure, i.e., two factors that determine the likelihood of a robot producing offspring. One is distance travelled. This is not an explicit objective, but it is implied by the fact that robots must come within communication range of eggs: robots that move about a lot have higher chances of meeting eggs and therefore procreate at a higher rate than robots that move little [25]. The second factor is the task performance that we explicitly introduced: when reviving, an egg selects a parent for the new controller based on the number of pucks collected. For a qualitative view of the importance of these two selective forces, consider Fig. 5. It plots the combined individuals of 64 runs in four 5,000 clock-tick intervals. Each individual is indicated by a small circle, of which the colour indicates the number of offspring for that individual. The position of the circle shows the number of pucks that individual collected (horizontal axis) and the total distance covered (vertical axis, in pixels in the simulated environment) during its lifetime.

Initially (left-most panel), there is little variation in terms of pucks collected (the dots are concentrated between 0 and 5 pucks collected). Individuals with high offspring counts are found across the range of distance travelled with a slight concentration between 500–1,000. This indicates that there is little pressure towards movement or collecting pucks at this point. As evolution progresses, at 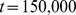, having offspring becomes contingent on travelling greater distances and collecting pucks; most robots travel substantial distances between 3,000 and 4,000 pixels. At 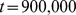, almost no individuals with more than one child that have travelled less than 2500 pixels. The two right-hand panels show that the number of collected pucks becomes increasingly important. These results indicate that robot behaviour initially adapts to the environment, evidenced by the initial differentiation in distance travelled. As evolution progresses and almost all robots travel substantial distances, the number of pucks collected becomes the dominant factor in determining the chances of producing offspring.

**Figure 6:**
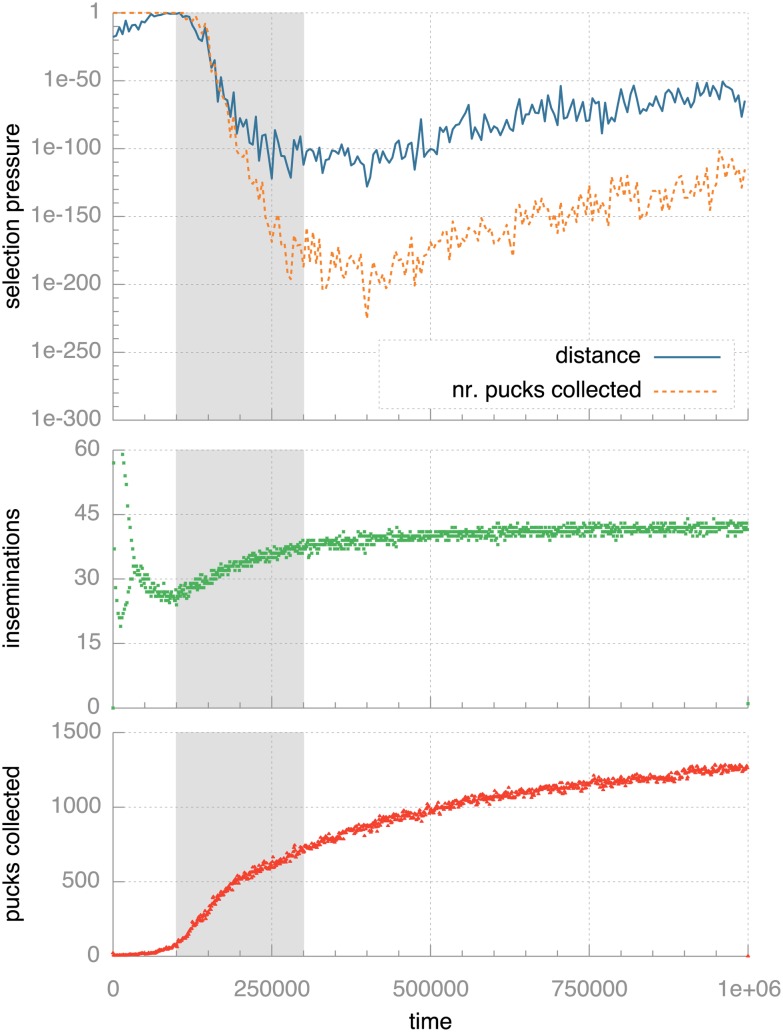
Quantitative analysis of selection pressure over time. The topmost graph shows how selection pressure develops over time. Selection pressure is quantified as the p-value from Fisher’s exact test: lower values indicate lower probability of random effects and so indicate higher selection pressure. Note the logarithmic scale on the vertical axis. The two plots below that repeat the data from Figs 4 and 3 for the standard monee experiments. The gray background highlights the period (between 100,000 and 300,000 ticks) where selection pressure rapidly increases at the same time that the population becomes successful at spreading genomes and collecting pucks.

For a quantitative analysis, we divide the experiment into slices of 5,000 ticks and then consider the robots that complete their lifetime during each interval as a population where we quantify selection pressure in terms of distance covered and pucks collected as described above. Just to recap, the p-values resulting from these tests indicate the probability that there is no relationship between having offspring and having above- or below-median distance travelled or pucks collected. Thus, low p-values indicate high selection pressure and vice versa. The top graph in Fig. 6 plots these values against time - the blue, solid line shows the p-values calculated on the basis of offspring and distance covered and the orange, dotted line shows the same for offspring and number of pucks collected. The graphs below that show the number of inseminations and number of pucks collected over the same time axis to allow visual comparison of trends in behaviour and in selection pressure (repeated from Figs. 4 and 3).

There is an initial, relatively low, selection pressure when regarding distance travelled (i.e., environmental selection pressure) and almost none in terms of pucks gathered (i.e., task-driven pressure). Then, the selection pressure starts to rise rapidly, first the pressure related to distance travelled, almost immediately followed by that in terms of pucks collected. From the insemination plot we can see that this rise coincides with a rapid increase in the number of inseminations, indicating that the population is getting to grips with the environmental demands for procreation (the initial peak in inseminations is caused by the fact that the robots are initially positioned close to each other). Just after the number of inseminations per time unit starts to level off, the selection pressure in terms of distance travelled as well as pucks collected reaches a high point: selection pressure is at its peak just as the number of inseminations starts to plateau. After that, selection pressure slowly reduces and seems to level off, presumably because the required behaviour is now so well established throughout the population that the relative evolutionary benefit of moving a lot and collecting many pucks is reducing. Selection pressure due to pucks collected and due to distance travelled follow similar trends, which is to be expected because collecting more pucks also implies travelling greater distance. The pressure from pucks collected outstrips that from distance covered after ca. 200,000 ticks and remains substantially higher. Because of the correlation between distance travelled and pucks collected, it is not possible to draw general conclusions about the relative selection pressure from environment and task with monee from these results.

While distance travelled and number of pucks collected have a strong impact on the chances of an individual procreating, there are three environmental parameters that affect the extent to which these two factors influence the likelihood of offspring. These parameters are:

**Communication range** How close to an egg must a robot be to be able to transmit its genome;

**Egg time** How long an egg is immobile and receptive to genomes before it revives;

**Life time** How much time a robot controller runs and has opportunity to disseminate its genome.

**Figure 7:**
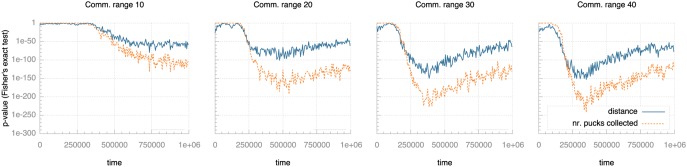
Selection pressure over time for different communication ranges. Selection pressure is quantified as the p-value from Fisher’s exact test: lower values indicate lower probability of random effects and so indicate higher selection pressure. Note the logarithmic scale on the vertical axis.

**Figure 8:**
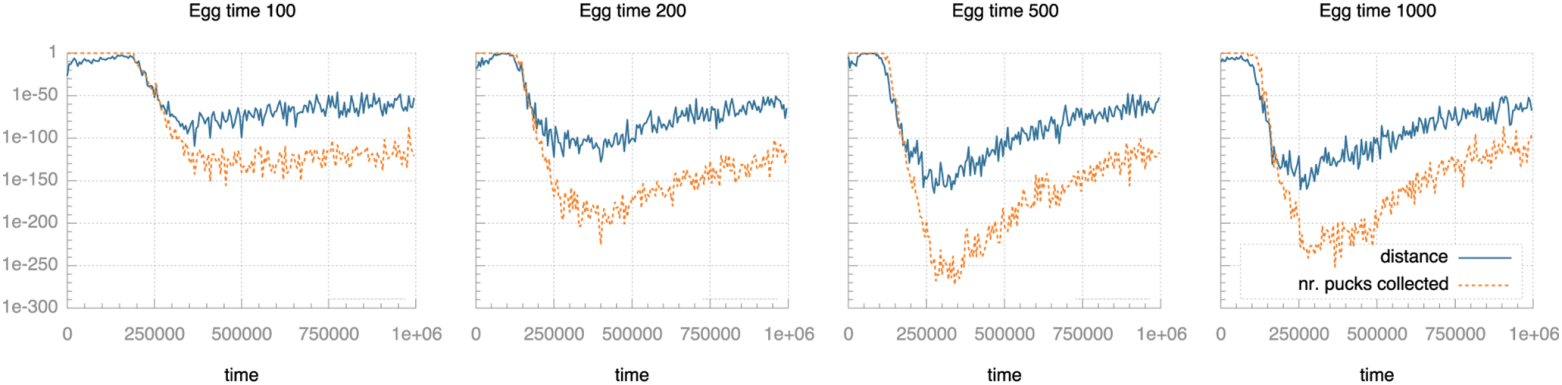
Selection pressure over time for different egg times. Selection pressure is quantified as the p-value from Fisher’s exact test: lower values indicate lower probability of random effects and so indicate higher selection pressure. Note the logarithmic scale on the vertical axis.

**Figure 9:**
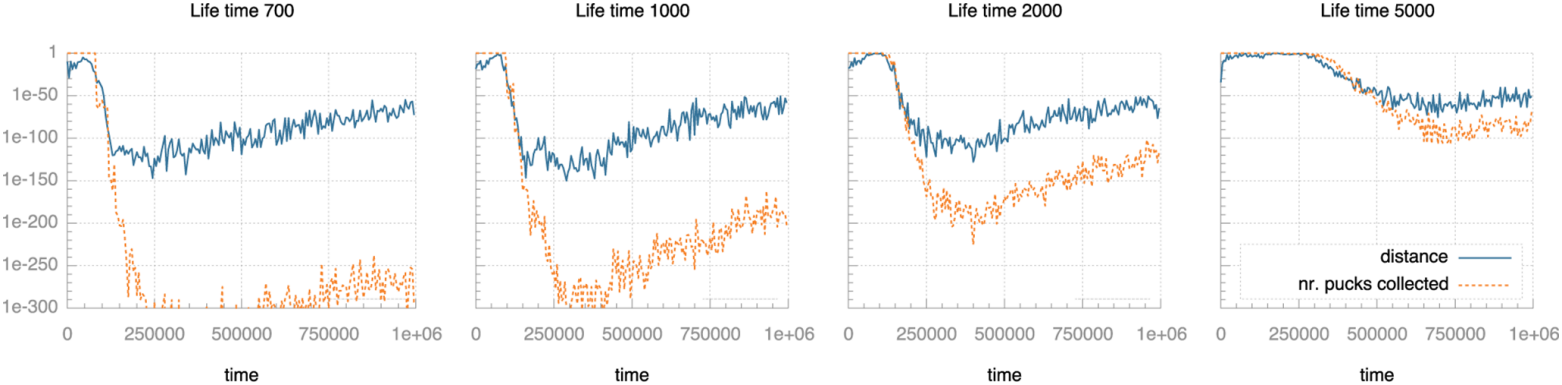
Selection pressure over time for different life times. Selection pressure is quantified as the p-value from Fisher’s exact test: lower values indicate lower probability of random effects and so indicate higher selection pressure. Note the logarithmic scale on the vertical axis.

We ran a number of experiments with different settings for these three parameters and measured the development of selection pressure as the experiments progress. Figures 7–9 plot selection pressure (calculated using Fisher’s exact test as described above) over time for different values of communication range, life time and egg time.

Figure 7 shows a profound impact of communication range on selection pressure. For short communication range (ca. one third body length), the pressure builds more slowly and to a substantially lower level than for medium communication (ca. one body length). Longer communication ranges increase the speed of build-up as well as the level of selection pressure, but this trend lessens for communication ranges over 30 pixels (greater than one body length).

Figure 8 shows that the peak selection pressure at ca. 300,000 ticks, particularly related to pucks collected, increases with egg time. Towards the end of the runs, the selection pressure has levelled off at similar levels for all settings, though, so the effect of egg time on selection pressure seems to be transient. It also seems that this increasing level of peak selection pressure no longer applies when increasing egg time from one quarter to one half life time (500 to 1,000). This may be because when the egg times are long enough, so many individuals are able to transmit their genome to the eggs that the in-egg selection procedure approximates panmictic selection. If that approximation becomes sufficiently accurate, further lengthening of egg time has no effect as each egg considers each genome only once: if an individual transmits its genome multiple times to an egg, the puck count for that genome is updated, but no additional entries are stored.

Analysing Fig. 9, we see a profound decrease in pressure from pucks collected as life time increases. The effect on pressure from distance covered is much more limited. Shorter life times mean that individuals that actively seek out pucks stand out more from those that merely move around and happen to collect pucks by doing so. This emphasised difference in task solving behaviour would explain the very high selection pressure for short life times.

### Distribution of Effort

The concurrent foraging scenario in our experiments implies a need to distribute the robot collective’s effort over the tasks: it is undesirable to have all robots collect pucks of only one colour. monee’s market mechanism was designed precisely to ensure an equitable distribution. It causes less commonly tackled tasks to reap higher rewards by introducing an exchange-rate per task.

**Figure 10:**
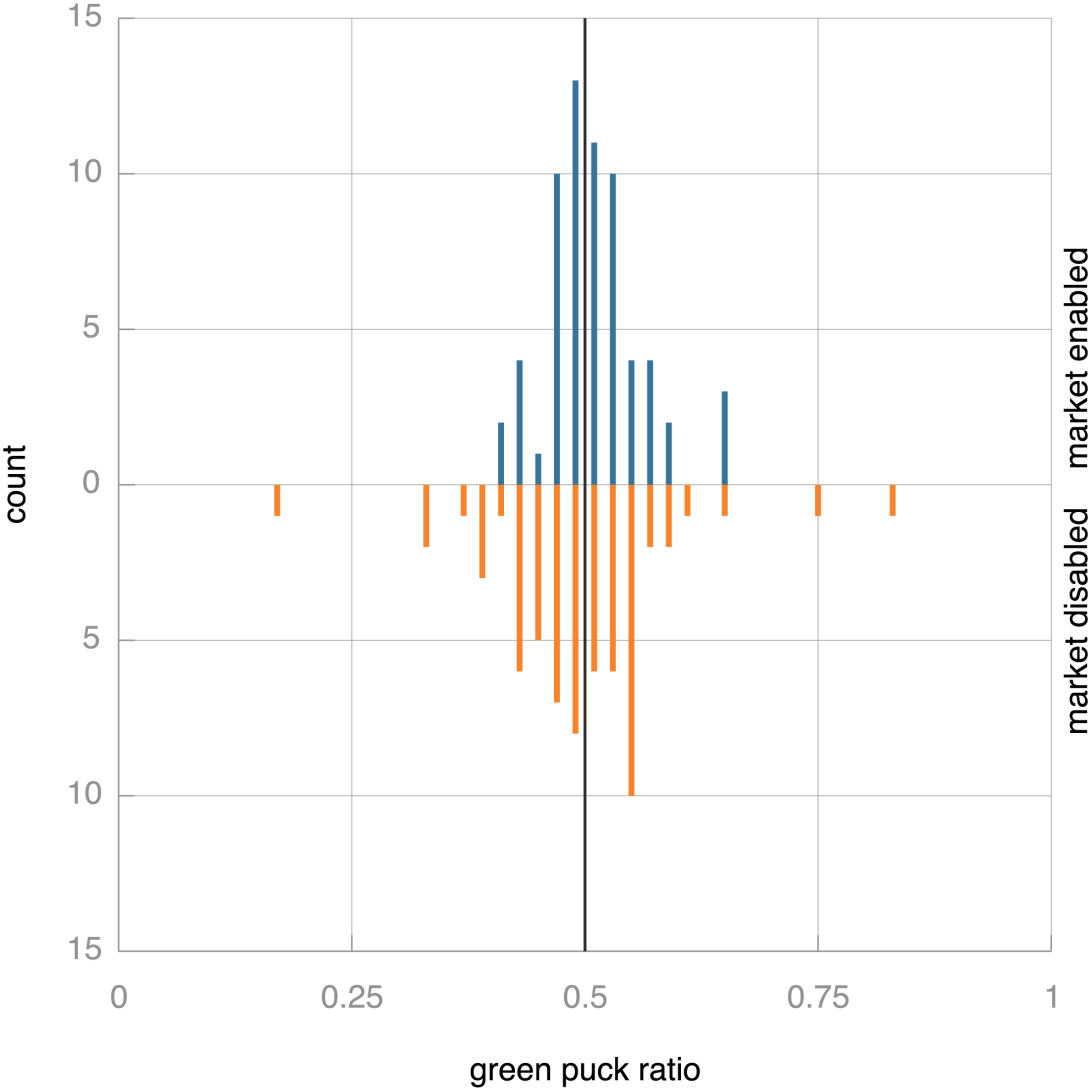
Bi-histogram of green puck ratios across the population with (top) and without (bottom) market mechanism over the final 1,000 time steps of simulation without environmental pressure towards specialisation. The environment contains equal amounts of red and green pucks, so equitable take-up of the tasks should result in a ratio of ca. 0.5, which would indicate that equal amounts of red and green pucks are collected. This ideal ratio is indicated by the black vertical line. The distribution with market mechanism seems tighter around the ‘natural’ ratio at 0.5. This difference is, however, not statistically significant at 5%: a two-sample Kolmogorov-Smirnov test to compare the distributions yields 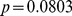.

To investigate whether the market mechanism does indeed provide for an equitable distribution of effort, we calculate the ratio between the number of green and red pucks collected by the population: this ratio should reflect the ratio in which the pucks are distributed throughout the environment. Thus, the percentage of green pucks collected indicates whether both tasks have been tackled equally successfully: an equitable distribution of effort would lead to populations where 50% (assuming equal numbers of green and red pucks in the environment) of the collected pucks is green. Figure 10 shows the distribution of the ratio of green to red pucks gathered by the populations in the final stages of the 64 runs of the standard experiment as well as in 64 runs of a control experiment where the market mechanism was disabled. In both cases the percentage of green pucks collected tends to the natural ratio of 0.5. With the market mechanism enabled, the distribution is more closely concentrated around this natural ratio than it is without the market, but the distributions are not significantly different with 5% confidence.

**Figure 11:**
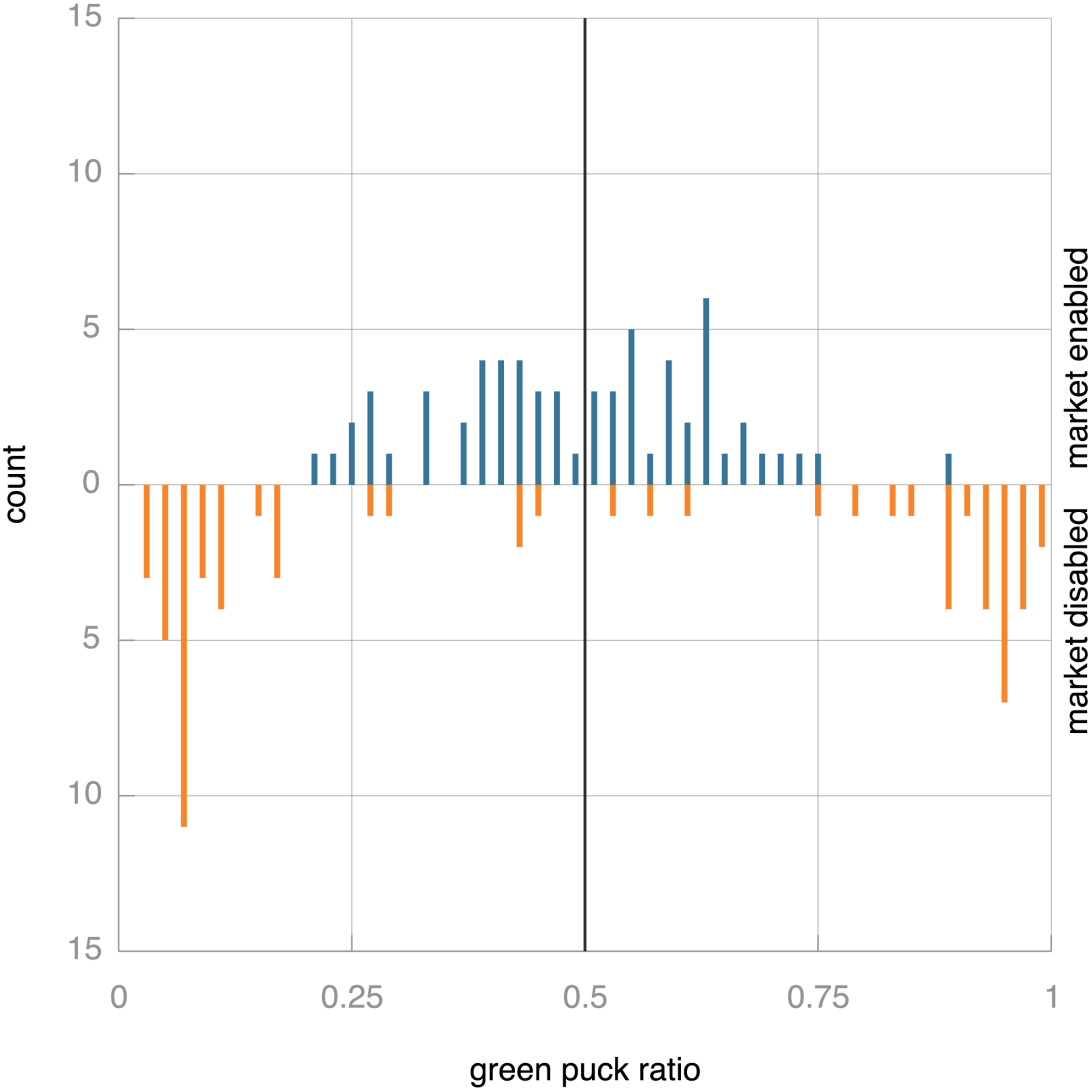
Bi-histogram of green puck ratios across the population with (top) and without (bottom) market mechanism over the final 1,000 time steps of simulation with environmental pressure towards specialisation. The environment contains equal amounts of red and green pucks, so equitable take-up of the tasks should result in a ratio of ca. 0.5, which would indicate that equal amounts of red and green pucks are collected. This ideal ratio is indicated by the black vertical line. Without the market mechanism, the robot collective tends to specialise in one type of puck, indicated by the two peaks near the extremes. A two-sample Kolmogorov-Smirnov test to compare the distributions yields 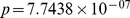.

In these experiments, the robots can collect green and red pucks equally well without any penalty when collecting both or merely one colour. To analyse what happens if the two tasks are (to some extent) mutually exclusive – i.e., if the robots are forced to specialise and focus on collecting only one colour of pucks–we introduce an incentive to specialise in the environment as follows. In the specialisation experiments the speed of robots depends on their specialisation level: the robot’s speed is multiplied by the ratio of most prevalent pucks it has collected: 
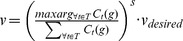
, with 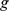 the currently activated genome and 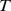 the set of all defined tasks. 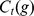 denotes the credits for task 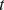 amassed with genome 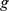. The specialisation level 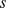 is set to 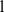 for this experiment. Thus, if a robot collects exclusively pucks of one colour, its speed is maximal. If it collects 75% green (or red) pucks, its speed is reduced by 25% and if it collects red and green pucks in equal amount, the speed is halved. This speed penalty is recalculated whenever a robot picks up a puck. Note that specialisation is enforced by the environment, not during the parent selection phase when an egg revives. The environment causes specialising robots to move faster, so that they perform better than non-specialised robots: their higher speed allows them to collect more pucks during their lifetime, but more importantly, it allows them to impregnate more eggs. Figure 11 shows that in such an environment the market mechanism is essential to keep the population from focussing on one task to the exclusion of the other. Although the distribution with market enabled is not as neatly focussed as it is in the multi-skilled setting, the population still collects both puck types in more or less equal amounts. Without the market mechanism, the majority of experiments resulted in a population that almost exclusively collects puck of one colour or the other. Rarely does the population gather even roughly the same number of green and red pucks.

**Figure 12:**
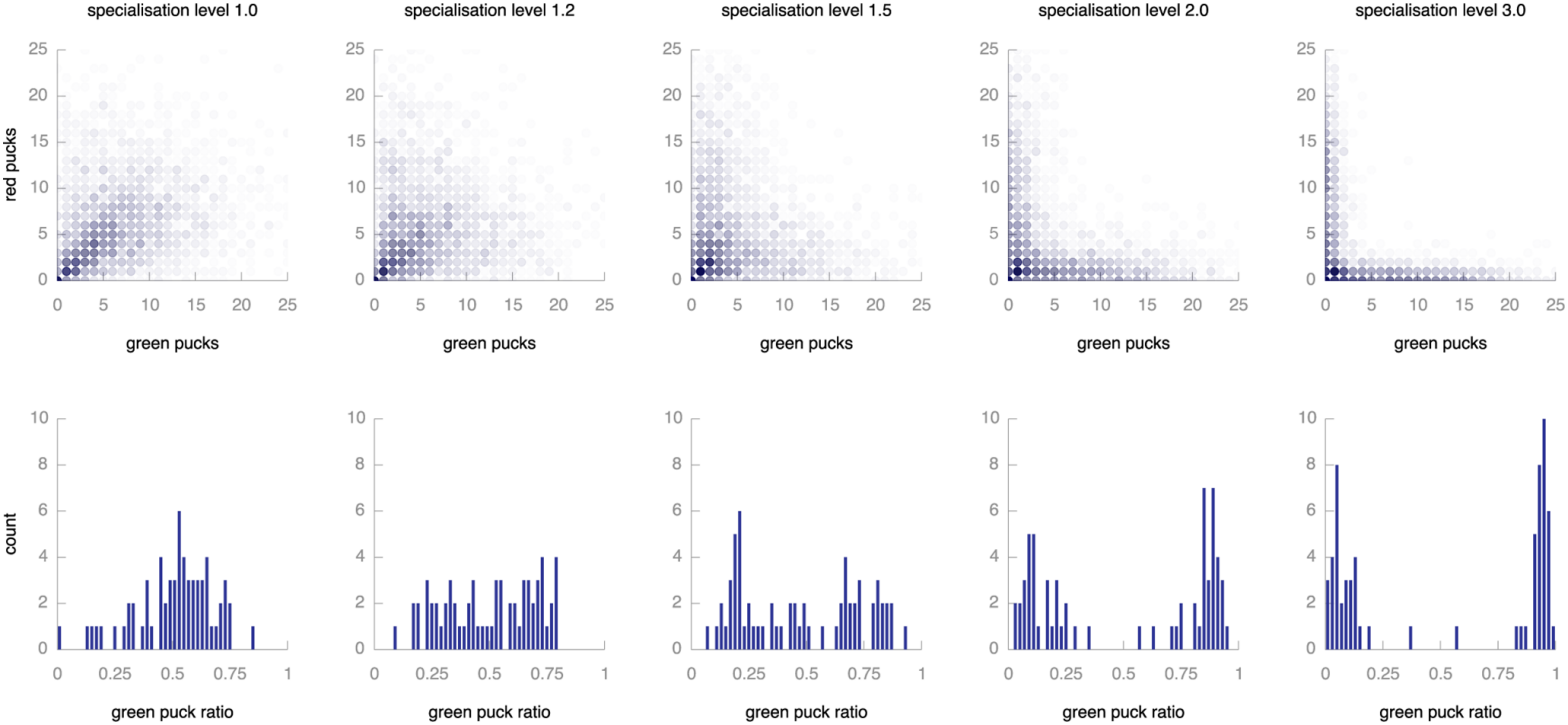
Effect of increasing pressure towards specialisation. The top row of graphs shows the level of specialisation of individual robots. Each circle represents a combination of red (vertical axis) and green (horizontal axis) pucks. Circles on the diagonal represent individuals that gather equal amounts of red and green pucks, circles on the axes represent specialised individuals. The colour intensity indicates the number of individuals (combined over 64 repeats of the experiment) that combined that particular combination during their lifetime. The bottom row contains histograms of green puck ratios across the population. Populations with a ratio of 0.5 are perfectly balanced, more extreme values indicate specialisation of the whole population on either type of puck. All plots report on the final 1,000 time steps of simulation.

Figure 12 shows results for runs with varying settings of the specialisation level 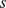. The top row plots show individual specialisation, the bottom row shows population level bar charts with the green puck ratio as a measure of specialisation as described above. We see that the market mechanism prevents population-level specialisation by promoting generalist behaviour at individual level. For lower specialisation levels, most individuals collect both types of puck in more or less equal measure (indicated by the dark dots on or close to the diagonal in the top plot). As we increase the pressure to specialise, individuals do focus on one type of puck: the darker dots are increasingly found at the edges. For these populations of specialising individuals, the market mechanism does not result in populations where two kinds of specialist coexist in balance and so fails to prevent overall specialisation when the environmental pressure to specialise is very high.

**Figure 13:**
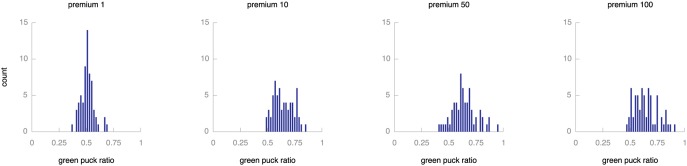
Effect of varying reward levels. The plot shows histograms of green puck ratios across the population for different premium values for green pucks. Populations with a ratio of 0.5 are perfectly balanced, more extreme values indicate specialisation of the whole population on either type of puck. All plots report on the final 1,000 time steps of simulation. Adding a premium factor to green pucks causes a slight preference for collecting green pucks, but this effect is not very large.

The two tasks in these experiments–collecting green and red pucks–are very similar. In particular, the credits for the two tasks compare trivially. More generally, however, it may be harder to determine comparable levels of credits for different tasks: how many credits should robots receive for monitoring resources, for collecting a certain amount or for transporting some resources some distance? To investigate the effect of different levels of reward for the tasks we run a number of experiments that define a premium for green pucks. Figure 13 shows the results of rewarding green pucks at 1, 10, 50 and 100 times as highly as red pucks. Again, we look at the distribution of green puck ratios towards the end of the experiment for 64 runs for each setting. The application of a premium to green pucks causes the population to collect more green than red pucks, but not to the extent that the red pucks are disregarded. Even with a premium factor as high as 100, the market mechanism causes the population to spend considerable effort on the red puck task. This implies that the relative value of different tasks has a limited effect on population behaviour. Consequently, there is no need to finely balance the rewards for different tasks: monee’s market mechanism ensures that all tasks remain in focus even if the reward levels differ substantially.

## Conclusions

This paper addressed the problem of mixing task-directed optimisation and environment-driven adaptation in the context of perpetual learning with a robot collective where communication is limited to local interaction. In particular, we argued that this needs to be resolved to enable the deployment of collectives of robotic agents in a real world situation: addressing user-defined tasks in open environments requires self-sustainability and a trade-off between optimal surviving strategies and addressing the tasks at hand.

The first important contribution of this paper is that we have shown that it is possible to combine objective-free environment-driven with task-driven evolution of behaviour in a population of simulated robots. The robots evolve behaviour that allows them to procreate in the environment as they do when no task is defined. monee introduces a second selection stage that takes task performance into account. Without compromising adaptation to the environment, this promotes behaviour that tackles tasks. In the set-up of our experiments, the tasks and the environment pose requirements that do not conflict with each other. Further research should investigate how this method of combining task- and environment-driven adaptation holds up in situations where task and environment conflict to some degree.

We investigated how selection pressure from the environment and the tasks develops over time. The results show that selection pressure rises steeply as the population experiences a rapid growth in effective behaviour from both an environment and a task perspective. After that, selection pressure eases as appropriate behaviour becomes prevalent and therefore less of a determinant of fecundity. Trying different settings for communication range, egg time and life time, we saw that these factors can substantially influence the development of selection pressure. The most profound difference occurred in the selection pressure for task-related behaviour when reducing life time values. Halving the life time increased selection pressure by orders of magnitude.

The second important contribution in this paper is the introduction of a market mechanism to efficiently balance collective effort over multiple tasks. When the robot collective is confronted with multiple tasks, the market mechanism compares rewards earned for the tasks and calculates an exchange rate to enable straightforward comparison of task performances. Our results show that this market mechanism promotes equitable distribution of collective effort over the tasks: both types of puck are collected in equal measure, even if there is some pressure for individual robots to specialise in one or the other. In our experiments, this distribution is achieved without detriment to performance: the number of pucks collected with market mechanism enabled is not statistically different to that without market mechanism. Generalist behaviour is effected at individual rather than at population level: individuals do not specialise as readily when the market mechanism is in force. The market mechanism does not maintain the distribution of effort when the pressure for specialisation is increased so far that individual robots *must* specialise. In that case, the population will most often focus on one of the defined tasks. Further research will have to investigate whether an amended market scheme or some additional method of distribution can guarantee an equitable distribution of effort under such circumstances.

In many situations, the tasks that the robots have to tackle will be more disparate than collecting two types of puck and it may consequently be hard to determine comparable reward levels for the different tasks. We simulated differently valued tasks by multiplying the rewards for collecting green pucks with a premium factor of up to 100. The results show that the market mechanism ensures if not an equitable at least a considerable share of collective effort devoted to the lesser valued task. This implies that it would not be necessary to determine exactly comparable reward levels, but getting them within one or even two orders of magnitude of each other could be good enough.

The monee paradigm that we introduced opens the door to significant further research: we feel that the successful combination of open-ended, survival-driven and objective-based, task-driven evolution is a crucial step on the road towards collectives of autonomous robots that can adapt to and operate effectively in unforeseen and dynamic circumstances. These two aspects of evolution combined can equip robot collectives with the adaptivity that coping autonomously with such uncertainty requires.
